# American Dental Association

**Published:** 1875-10

**Authors:** 


### SELECTED ARTICLES.

---

### ARTICLE III.

### *The American Dental Association—Fifteenth Annual Session.*

FIRST DAY—*Morning Session.*

The fifteenth annual session of the American Dental Association convened at Grant's Hall, Niagara Falls, on Tuesday, August 3d, 1875 ; the president, Dr. M. S. Dean, of Chicago, in the chair.

The usual routine business of roll call and reading of minutes being disposed of, and a partial report of the Committee on Credentials having been made, miscellaneous business was taken up, and several proposed amendments to the constitution, resolutions, etc., lying over from last year, were acted upon.

The first of these, an amendment proposed by Dr. Shepard, giving the Executive Committee the power to change the place of meeting for good reasons, was, after discussion withdrawn by the mover.

Two others, proposed by Dr. Cushing,—the first making a change in the manner of appointing a Committee of Arrangements, providing for their appointment by the President, instead of being a division of the Executive Committee, —were taken up and passed.

An amendment by Dr. Stockton, providing for a change in the manner of electing officers, was laid on the table.

An amendment proposed by Dr. Allport was then taken up, which read as follows:

This association will hereafter receive no delegate who shall, after this date, enter the profession without first having graduated at some reputable dental or medical college.

This proposition gave rise to considerable discussion. Dr. Atkinson and others thought it signified a tendency toward exclusiveness, while Drs. Rehwinkel, Morgan, and others thought it did not go far enough , and Dr. Morgan proposed a substitute, which provided that no delegate should hereafter be received who did not hold a diploma, thus going further than the original amendment, which only applied to those who might *hereafter enter* the profession.

Dr. Morgan subsequentlly, by consent, withdrew the substitute, and the original amendment was then put and carried.

[Local societies sending delegates to the American Dental Association will therefore bear in mind that no person coming into the profession after this date can be received as a delegate unless he be a graduate in medicine or dentistry.]

An amendment, offered by Dr. McQuillen, changing the number requisite for a quorum from fifteen to twenty-five, was lost.

Some resolutions on the subject of "Dental Education," lying over from last year, were then taken up.

One, offered by Dr. Bogue, as follows:

*Resolved*, That it is the sense of this association that no dental student should be graduated from any dental college without at least three years' instruction; the latter should in no case embrace less than two full courses, was, after discussion adopted.

Another, by the same person, suggesting a common committee, to be appointed by the colleges, but disconnected with any of them, for the purpose of examining candidates for graduation and deciding upon the same, was voted down as impracticable.

A resolution on the same subject, offered by Dr. Judd, was taken up and adopted, as follows:

*Resolved*, That this association recommends to all local societies the adoption of rules prohibiting their members from taken students for a less period than three years, or for such time as will complete a three years' pupilage.

An invitation to visit certain objects of interest was accepted.

A resolution extending to Dr. Wilson, of Cuba, and any others who might be present from foreign countries, the privilege of the floor, was adopted.

After some further routine business, reports of committees were taken up; but those called not being ready, the meeting adjourned till 7.30 P. M.

### *Evening Session.*

The association was called to order at 7.45, by the Presdent.

The subject of "Physiology" was in order; but the chairman of the committee not being present, it was moved by Dr. H. A. Smith, and carried, that Dr. John Allen be invited to read a volunteer essay upon the subject.

Dr. Allen's paper was upon the subject of physiology as applied to the occupation and health of the dentist.

He alluded to the fact that the duties of the dentist were increased in proportion to the degree of eminence we have attained. No profession more severely taxes the mind, brain, and nervous system than our own, and one hour of the required tension often tells upon the dentists more than would a day of ordinary toil. The temperament of patients often exhausts his vital forces rapidly.

Now, it is important to consider how we may prevent a premature exhaustion of the vital forces. Narcotics, stimulants, exercise, have been restorted to. The more careful we are to observe the fixed laws of nature the more we receive strength.

The human body with its numerous organs and tissues is built up, atom by atom, from fourteen nutrient substances, derived from the food, They are divided into four classes, viz., proteids, fats, amyloids, and minerals. Men introduces other substances which are non-nutrient and detrimental. These are narcotics,-tobacco, alcohol, and opium. They are poisons, but have their places, judiciously used, as medicines; but in larger doses produce narcosis, coma, and death. When only the proper materials in due proportion are introduced into the system, the equipoise of waste and supply is maintained; but narcotics and stimulants do not restore the vital forces. To what is the peculiar fatality in our ranks owing? Is it to the exhaustive nature of our labors, or a course of habits involving the use of these articles? or is it the want of daily excercise in the open air?

We recommend that this subject be brought before the State and local societies, and to place the matter in a tangible shape, the paper closed by the introduction of the following resolution:

*Resolved*, That a committee of three be appointed, whose duty it shall be to collect facts with reference to the subject matter here presented, and also the best means of preserving the physical and mental capacity of dental practitioners.

The resolution was put to vote and carried.

[The Chair subsequently appointed as the committees provided for by this resolution Drs. John Allen, W. H. Morgan, and W. E. Magill.]

Dr. McQuillen, chairman of the committee, then read a report on "Sleep, Dreams, and Anæsthesia."

The subject being open for discussion, Dr. Atkinson said that he rejoiced that we were apparently approaching a solution of principles. All that we have had has been description of condition. Sleep has not been defined. It has been assumed that we know what it is. Without a definition of it we can arrive at no profitable conclusion as to the difference between sleep and anæsthesia. Can I point out the difference between sleeping and waking? If I should say that it was simply polarization and depolarization, how many would understand me? We are dealing with something not quite so tangible as a stone fence when we treat of a subject like sleep.

There is resident within the organism a demand for a sufficient quantum of power to operate the functions of the body; so long as that demand is supplied and has a credit account in the bank, we are waking. In the practice of medicine he has known no sleep for weeks as generally understood, and has slept in the saddle; is subject to neuralgia, and must have a plus quantity of force in his system not to feel constant pain. These details are difficult to explain to those not familiar with the generation of the elements of tissues. Chemistry is a mere maze of scapegoats to enable us to appear learned when we are the merest blanks. The alphabetical nomination of modes of motions is the only method of ascertaining what physiology is. We must have perception before we can formulate. If we say that sleep and anæsthesia are caused by congestion of the blood-vessels, we show dense ignorance, since we attempt to pronounce definitely upon what we have only insufficient means of determining. *Anæsthesia is death*, in the ratio of its manifestations.

What are poisons? Agents said to act on nerve-centers! The first nerve-center is only a bleb of nervous mass. A human being is only a protoplasm. Oxygen is not a supporter of combustion till set free, and we all know it is then.

Is anæsthesia sleep? A kind of sleep. What is the difference between sleep and death? One is a depolarization forever; the other is only temporary, and is not so in relation to breathing. If we slept all over at once we would die all over at the same time. How does the heart get along? Does it sleep? and do the lungs sleep?

Dr. McQuillen. The theory that the blood-corpuscle consists of a vesicle or cell wall with fluid contents, as stated by the preceding speaker, has long since been abandoned by the majority of histologists. It is now generally regarded as a soft-solid substance. The heart and lungs do sleep, rest or recuperate. They sleep in the regularly recurring intervals between the beatings and the respirations. Place the ear over the chest, and two sounds made by the heart will be heard—a long, dull sound, and a short, quick sound, followed by an interval of repose. The heart rests or sleeps six hours out of the twenty-four. If it be assumed with Dr. Snow, that anæsthetics do not act directly upon the nerve-centers as a poison, but that anæsthesia is due to privity of oxygen, it must follow that the inebriate reeling through the streets is not drunk, but is only suffering from privity of oxygen. Shall it be said that sleep is caused by privity of oxygen? Why, during the waking period the quantity of oxygen consumed in the act of respiration is far in excess of that used during sleep. It is difficult to define what sleep is. The brain fails to comprehend and language fails to express these subtle physiological principles. We can only describe phenomena; their causes often lie too deep for the mind to grasp. Physiologists discover, as did Columbus sailing into unknown seas. Scientists working in new fields are liable to make mistakes.—but blunders sometimes lead to success, though often not in the direction intended.

Dr. Atkinson. It *is* privity of oxygen, and nothing else, which makes these men drunk. Don't let us call things by the wrong names. NO is almost always $NO^2$, and whatever amount of this may be inhaled satisfies the affinity of the blood-corpuscles for oxygen. Even when in cylinders the very purest that can be made, it contains $NO^2$, and do not let us be fooled into believing anything else. When it comes into contact with a blood-corpuscle it is satisfied, and then there is effete matter. This is the foundation of many diseases, such as tubercle, etc.

Dr. Magill. The practical questions to be drawn from these papers are: What induces insomnia? Is it mental excitement, or the tension under which we labor? We are all conscious of exhaustion, and Dr. Allen opens a practical view of the subject. We should seek the remedy in relief from excitement. We should discuss whether sleep is the withdrawal of blood from the brain; if so, a remedy which has been suggested for insomnia, namely, eating to induce a flow of blood to the stomach, and consequent withdrawal of it from the brain, may be founded in reason. We suffer from extreme exhaustion, and anything which will bring us to understand the causes of, and so prevent our trouble, will be better than any remedy.

Prof. Flagg. Practical results are what we want; unless something practical can be drawn from these papers and discussions, no tangible result will be reached. Sleep is an extended subject, and the speaker had done a great deal of it in his time, and expects to do more, and feels competent to discuss it. (Laughter.) In that part of his remarks which I could understand, Dr. Atkinson seems to have struck near the mark, and it is natural for me to infer that the still larger portion that I could not understand was still sounder. (Laughter.)

Sleep is the desire on the part of the organism for further life. As it is deep and strong it is recuperative; as it is recuperative; as it is short, slight, and disturbed, it is dissatisfying, it is our duty to recognize that we become exhausted mentally, physically, and, we may almost say, *morally;* we ought to go to bed early and sleep enough. Don't get upearly and work till night over the chair, and then go into the laboratory till midnight, and then go to reading up. The speaker had become entirely prostrated by hard work and reduced to one hundred and twenty pounds; he had changed his mode of life, living eight miles from his office, and never going near it on Saturdays, (thus doubly fulfilling the Biblical injunction, which only commands one day's rest,) and by this change, and eating the best food he could get, and sleeping eight hours every night, had increased his weight to one hundred and sixty pounds.

Anæsthesia is death? Because it does not prove fatal every time, do not let us think that it is not harmful. It sows disease; it produces a condition favorable to such diseases as chorea and other nervous affections, which will unfit for business, society, or any of the active duties of life. The asylums are full of cases of these diseases. The victims will tell you that from the time of taking ether they began to have their symptoms; first a little irritation of the muscles of the throat, then barking, and then twitching Men are innocently (rather than ignorantly) swung down to the jaws of death and back again. We have to do it, but it is little less than death. The patient is never the same after. He knows of what he speaks, for he has administered anæsthetics from three to five times a day for twenty years, and every time he did it he trembled for the patient under his care. The effect on himself was fearful—insomnia, nervous irritability, etc.—until he abandoned it.

Prof. Taft wishes to emphasize the expressions of Prof. Flagg. It is an important subject and a serious question. A large number of the profession are in the constant habit of using anæsthetics; many use mostly nitrous oxide: If the influence specified is liable to occur to the patient, how must it be with the operator who is constantly administering it? Prof. Flagg tells his own story; the speaker knows of instances where the health of operators has been injured. This accounts for the cases of insanity. The exhausting nature of the work does not all depend on prolonged effort but is largely due to incompatibility between operator and patient. One individual will exhaust the strength more in an hour than another will in a day. The strength and mental energy are taken away in such cases, but in others they are apparently increased. He refuses to operate for some patients on that ground alone.

Dr. Rehwinkel bears testimony to the truth of the statements of Profs. Taft and Flagg. He formerly served a term in the penitentiary (laughter,) and while there looked upon chloroform as harmless as water. It was in 1847 and 1848, and the Government had given orders that the new agent should be thoroughly tested. The anatomical students were in want of material, and it was hinted that no responsibility would be incurred in case of accident. The consequence was that not a hair was pulled out with chloroform; cases were hunted up among the convicts, who were ready to submit, it being a pleasing variety to them. He had come very near expediting many patients to the happy land. The more he used the agent the more he became afraid of it, and for the last ten years had only consented to use it in cases where the chances of life and death were evenly balanced. He spoke of a singular case in Germany: A gentleman of a poetic nature having read of the stimulating and exhilarating effects of ether, procured a bottle, and putting himself under its influence, experienced results similar to those arising from hasheesh-eating; he continued its use, but did not find a recurrence of his delightful visions. He became, however, a confirmed ether-taker, consuming as much as two pounds a day; he expended his means to procure ether, and when he had no more he became the terror of druggists. He would buy it or beg it, and would have stolen it if he dared. He was finally admitted to a hospital, and a test made to see how much it would take to plaee him under the influence ot ether; with an improved inhaler, which allowed no air to enter, six and three-quarter ounces were consumed before he was partially etherized! Adjourned.

TO BE CONTINUED.

